# Cytosolic Delivery of Thiolated Neoantigen Nano‐Vaccine Combined with Immune Checkpoint Blockade to Boost Anti‐Cancer T Cell Immunity

**DOI:** 10.1002/advs.202003504

**Published:** 2021-01-29

**Authors:** Da Zhang, Ziguo Lin, Ming Wu, Zhixiong Cai, Youshi Zheng, Lei He, Zhenli Li, Jie Zhou, Liqin Sun, Geng Chen, Yongyi Zeng, Juan Li, Jingfeng Liu, Huanghao Yang, Xiaolong Liu

**Affiliations:** ^1^ The United Innovation of Mengchao Hepatobiliary Technology Key Laboratory of Fujian Province Mengchao Hepatobiliary Hospital of Fujian Medical University Fuzhou 350025 P. R. China; ^2^ The Key Lab of Analysis and Detection Technology for Food Safety of the MOE, Fujian Provincial Key Laboratory of Analysis and Detection Technology for Food Safety, College of Chemistry Fuzhou University Fuzhou 350002 P. R. China; ^3^ Mengchao Med‐X Center Fuzhou University Fuzhou 350116 P. R. China; ^4^ CAS Key Laboratory of Design and Assembly of Functional Nanostructures, Fujian Institute of Research on the Structure of Matter Chinese Academy of Sciences Fuzhou 350002 P. R. China

**Keywords:** cytosolic delivery, immune checkpoint, neoantigen, T‐cells immunity, thiolated nano‐vaccine

## Abstract

Although tumor‐specific neoantigen‐based cancer vaccines hold tremendous potential, it still faces low cross‐presentation associated with severe degradation via endocytosis pathway. Herein, a thiolated nano‐vaccine allowing direct cytosolic delivery of neoantigen and Toll like receptor 9 agonist CpG‐ODN is developed. This approach is capable of bypassing the endo‐/lysosome degradation, increasing uptake and local concentration of neoantigen and CpG‐ODN to activate antigen‐presenting cells, significantly strengthening the anti‐cancer T‐cell immunity. In vivo immunization with thiolated nano‐vaccine enhanced the lymph organ homing and promoted the antigen presentation on dendritic cells, effectively inhibited tumor growth, and significantly prolonged the survival of H22‐bearing mice. Strikingly, further combination of the thiolated nano‐vaccine with anti‐programmed cell death protein‐1 antibody (*α*PD‐1) could efficiently reverse immunosuppression and enhance response rate of tumors, which led to enhanced tumor elimination, complete prevention of tumor re‐challenge, and long‐term survival above 150 d. Collectively, a versatile methodology to design cancer vaccines for strengthening anti‐cancer T‐cell immunity in solid tumors is presented, which could be further remarkably enhanced by combining with immune checkpoint inhibitors.

## Graphical abstract

A thiolated nano‐vaccine allowing direct cytosolic delivery of neoantigen and Toll like receptor 9 agonist CpG‐ODN is reported. This approach is capable of bypassing the endo‐/lysosome degradation, increasing uptake and local concentration of neoantigen and CpG‐ODN to activate antigen‐presenting cells, significantly strengthening anti‐cancer T‐cell immunity in solid tumors, which could be further remarkably enhanced by combining with immune checkpoint inhibitors.

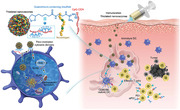

## 1 Introduction

Hepatocellular carcinoma (HCC) is the fourth most common cause of cancer‐related death worldwide.^[^ ^1^ ^]^ Traditional treatments for HCC, including surgery, radio‐chemotherapy, and targeted therapy, showed a great potential but generally achieved poor prognosis in clinic.^[^ ^2^ ^]^ It is due to the tumor heterogeneity and the high density of HCC tumors with trabecular, compact, pseudo‐glandular, and sclerosing variants intermingled throughout the tissue, which also extremely restricted the efficiency of drug delivery into the HCC tumors.^[^ ^3^ ^]^ Cancer immunotherapy has become one of the most promising therapeutic approaches for cancer treatment through harnessing and boosting the patient's own immune system to eliminate both the primary and metastatic tumor cells.^[^ ^4^, ^5^, ^6^, ^7^ ^]^ However, the objective response rate of immunotherapy in HCC, including checkpoint inhibitors such as programmed cell death protein‐1(PD‐1) or programmed cell death protein‐1 ligand (PD‐L1) antibodies and chimeric antigen receptor T‐cell therapy, is still extremely limited due to the weak immunogenicity of “cold” tumors with restricted tumor‐infiltrating lymphocytes (TILs)^[^ ^8^, ^9^ ^]^

Recently, tumor‐associated antigens (TAAs), which are frequently overexpressed in tumor tissue, have been proved as candidates for personalized cancer vaccines (PCVs) in cancer immunotherapy.^[^ ^10^, ^11^, ^12^ ^]^ But their induced immune responses were still disappointing because of the existence of self‐tolerance in immune system. In contrast, tumor neoantigens derived from expressed specific genome somatic mutations in tumor tissues have been actively developed as attractive targets for cancer immunotherapy; these specific non‐self‐peptides screened by computational pipeline are tumor specific antigens without pre‐existing central tolerance, and could induce much stronger immunogenicity for intensively activating T cells to effectively destroy tumor cells and inhibit tumor progression comparing with classical TAAs.^[^ ^12^ ^]^ Accordingly, the feasibility of neoantigen based immunotherapies such as long‐peptide vaccines, dendritic cell (DC) vaccines, and neoantigen‐reactive T cells have been extensively verified in mice and patients.^[^ ^13^, ^14^, ^15^ ^]^ Nevertheless, due to the low expression of mutational burden in tumors, the limited numbers of neoantigens need to be more efficiently cross‐presented to induce a more robust and effective anticancer T cell response for cancer treatments.^[^ ^16^ ^]^ Regrettably, the therapeutic outcomes of the vast majority of neoantigen vaccines in clinical trials still remain disappointing due to their low cross‐presentation efficiency by antigen presenting cells (APCs) mainly attributing to the degradation of neoantigens/adjuvants in lysosomes and insufficient lymph node (LN) homing.^[^ ^17^, ^18^, ^19^, ^20^ ^]^

Cytosolic delivery of neoantigen into APCs, for example, DCs, has been demonstrated to be an effective strategy to augment the cross‐presentation efficiency by lowering down the degradation of antigens/adjuvants to improve immune responses.^[^ ^21^, ^22^, ^23^, ^24^, ^25^ ^]^ Recently, nano drug delivery formulations have offered the potential to specifically accumulate in LNs and “on‐demand” release inclusions to target cells at the right time and right site.^[^ ^20^, ^24^, ^26^, ^27^, ^28^, ^29^, ^30^ ^]^ Thus, developing a novel cytosolic delivery strategy based on nanosystem would be extremely attractive for enhancing the cross presentation to augment its cancer immunotherapy efficiency. Furthermore, due to the existence of tumor immunosuppressive microenvironment, immune checkpoint blockade (ICB) on T cells such as the PD‐1 antibody or cytotoxic T‐lymphocyte associated protein‐4 antibody has been considered as an efficient strategy to enhance anti‐tumor T‐cell immunity and prevent tumor recurrence and metastasis in pre‐clinic or clinic practices.^[^ ^31^, ^32^, ^33^ ^]^ Therefore, combination of neoantigen based PCV with ICB might be a promising synergistic regimen to enhance immunogenicity, augment LN homing, and increase TILs, which may lead to a new era in HCC immunotherapy.

**Figure 1:**
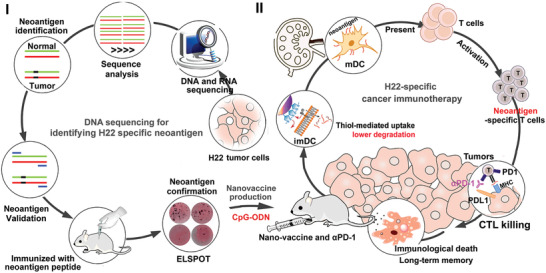
Schematic illustration of the neoantigen screening process, the preparation of thiolated nano‐vaccine, and the mechanisms for personalized cancer immunotherapy. The H22 liver cancer cell specific neoantigens were screened by performing whole‐exome sequencing and transcriptome sequencing, and the potential neoantigens were predicted by in silico analysis and their immunogenicity were confirmed by ELISPOT assay. Afterward, the thiolated nano‐vaccine was prepared by self‐assembly of guanidinium (Gu^+^)‐containing disulfide monomers, CpG‐ODN and selected neoantigen through electrostatic interaction and *π*–*π* stacking. After i. p. injection of nano‐vaccine, it could be highly efficient uptaken by imDCs to drastically improve the codelivery efficiency of adjuvants and neoantigens to LNs, and subsequently promoting neoantigen‐specific CD8+T cell proliferation, which leads to efficient suppression of tumor growth and elimination of established tumors, prevention of tumor re‐challenge, and thereafter prolongation of survival rate and survival time of HCC tumor‐bearing mice.

Herein, a bottom‐up design strategy was developed to construct the guanidinium‐containing disulfide (Gu^+^‐unit) based thiolated nano‐vaccine through assembly of the Toll‐like receptor 9 (TLR9) adjuvant CpG‐ODN and a mouse liver cancer cell specific neoantigen (predicted by in silico analysis and verified through enzyme‐linked immunospot assay (ELISPOT)) to co‐deliver the antigen/adjuvant directly into cell cytosol by thiol‐mediated cellular internalization pathway for enhancing the cross‐presentation efficiency by APCs (**Figure** **1**). In the design, the Gu^+^‐unit acted as a bridge to assemble with CpG‐ODN (20 nt) and neoantigen (17 mer); upon immunization with this thiolated nano‐vaccine, the so‐called thiol‐mediated cytosolic pathway could significantly increase neoantigen and CpG‐ODN local concentrations in APCs, leading to a robust T cell immune response to prevent the tumor growth and prolong the survival time of H22 tumor‐bearing mice. Furthermore, in combination with ICB antibody (*α*PD1) treatment, the thiolated nano‐vaccine enabled almost complete elimination of established primary tumors, efficiently prevented tumor re‐challenge, and significantly prolonged the survival of H22 tumor bearing mice with more than 150 d through inducing robust immune responses and long‐term immune‐memory. Collectively, this study offers a promising cytosolic direct delivery strategy to design PCVs for strengthening anti‐cancer immune responses, which might hold a great potential for future clinical translation in HCC immunotherapy.

## 2 Results

### 2.1 Screening HCC‐Specific Neoantigens and Construction of Thiolated Nano‐Vaccine

**Figure 2:**
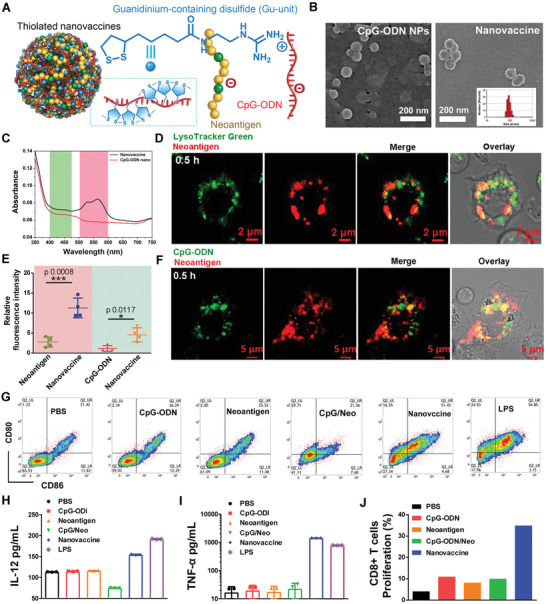
Characteristics and immuno‐stimulation of thiolated nano‐vaccine. A) Structure and intermolecular interaction of Gu^+^‐unit based thiolated nano‐vaccine. B) TEM image of CpG‐ODN NPs and thiolated nano‐vaccine. The insert picture presented the DLS distribution of thiolated nano‐vaccine. C) The Uv–vis absorbance of CpG‐ODN NPs (CpG‐ODN^FAM^) and thiolated nano‐vaccine (CpG‐ODN^FAM^/neoantigen^Cy3^). D) The sub‐cellular location of thiolated nano‐vaccine (CpG‐ODN/neoantigen^Cy3^) in BMDCs after 0.5 h incubation and lysosome staining with LysoTracker Green. E) Relative fluorescence intensity of thiolated nano‐vaccine (CpG‐ODN^FAM^/neoantigen^Cy3^), CpG‐ODN^FAM^, and neoantigen^Cy3^ in bone marrow‐derived dendritic cells (BMDCs) for 0.5 h incubation. Results are shown as mean ± SD. (*n* = 4). F) Cellular uptake of thiolated nano‐vaccine (CpG‐ODN^FAM^/neoantigen^Cy3^) after co‐incubation with BMDCs for 0.5 h. G) The maturation of BMDCs after co‐incubation with CpG‐ODN, neoantigen, CpG‐ODN/neoantigen mix, thiolated nano‐vaccine, and LPS for 72 h, respectively. The secretion of H) IL‐12 H and I) TNF‐*α* was further checked by ELISA assays at 72 h of post‐incubation. Results are shown as mean ± SD. (*n* = 3). J) The proliferation of CD8+T cells after co‐incubation with matured BMDCs for 48 h.

The potential specific neoantigens in H22 cells (mouse liver cancer cells) were screened by in silico analysis of whole‐exome sequencing data and transcriptome sequencing data of H22 cells, and further confirmed by ELISPOT assay (Figure S1, Supporting Information). Afterward, the high immunogenicity H22 tumor cell‐specific neoantigen consisting of 17 amino‐acid sequences (HTDAHAQAFAALFDSMH) with reasonable pI (5.71) (evaluated by ExPASy) and negative charge was self‐assembled with CpG‐ODN and Gu^+^‐unit (prepared by our previously reported work^[^ ^34^ ^]^ via the electrostatic interaction between the phosphate groups (PO_4_^−^) of CpG‐ODN and the Gu^+^ units to form the thiolated nano‐vaccine (**Figure** **2**A). After shaking for 15 min (the shaking and incubation time of Gu^+^ units and CpG‐ODN/neoantigen was optimized and then confirmed by polyacrylamide gel electrophoresis (PAGE)), the thiolated nano‐vaccine was obtained and showed uniform size distribution of 85.2 ± 8.6 nm, as confirmed by SEM and DLS analysis, and it was larger than CpG‐ODN/Gu^+^ units‐assembled nanoparticles (CpG‐ODN NPs) which had a diameter of 55.3 nm ± 9.1 nm (Figure 2B; Figures S2 and S3, Supporting Information). The zeta potential of our thiolated nano‐vaccine was −10.7 mV, comparing to Gu^+^ unit with +47.8 mV, attributing to the negative charge of oligonucleotides and neoantigen (Figure S4, Supporting Information). UV–vis‐NIR absorbance of thiolated nano‐vaccine exhibited two typical absorption peaks of dyes at 450 nm (FAM labeled CpG‐ODN) and 560 nm (Cy3 labeled neoantigen), corresponding with their fluorescence spectra, indicating successful assembly of neoantigens and CpG‐ODN inside the thiolated nano‐vaccine (Figure 1C and Figure S5, Supporting Information). PAGE profile of the thiolated nanovaccine after incubation with FBS 10% at 4 °C in neutral solution for 48 h showed no obvious degradation, demonstrating its bio‐stability (Figure S6, Supporting Information).

### 2.2 Enhancement of Neoantigen Cross‐Presentation Efficiency and BMDCs Maturation

The cross‐presentation efficiency by APCs determines the anti‐cancer T cell immune responses.^[^ ^35^ ^]^ According to our and previous reports of counterion‐thiol‐mediated uptake mechanism,^[^ ^34^, ^36^ ^]^ the thiolated nanovaccine could first bind to the cell membrane and then bypass lysosomal degradation through counterion‐mediated transient micellar pores, which might augment the cross‐presentation efficiency by rat bone marrow‐derived DCs (BMDCs). To this end, confocal microscopy (CLSM) was conducted to evaluate the intracellular release of CpG‐ODN^FAM^ and neoantigen^Cy3^ in BMDCs. After incubation with the thiolated nano‐vaccine (CpG‐ODN, neoantigen^Cy3^) for 0.5 or 2 h, clear separation of the red fluorescence signal of thiolated nano‐vaccine (neoantigen^Cy3^) and the green fluorescence signal of lysosome stained by LysoTracker Green was observed. And the Pearson's correlation coefficients were determined to be 0.069 (0.5 h) and 0.13 (2 h), respectively, which were far below the threshold of >0.5, verifying that the uptake of thiolated nano‐vaccine bypassed lysosome pathway (Figure 2D and Figure S7, Supporting Information). Moreover, strong green fluorescence signals of FAM from CpG‐ODN^FAM^ and red fluorescence signals of Cy3 from neoantigen^Cy3^ could be clearly observed and predominantly localized in the cytosol of BMDCs after only 0.5 h incubation. In addition, the fluorescence signals of free CpG‐ODN^FAM^ or neoantigen^Cy3^ alone was much lower than those manifested by the thiolated nano‐vaccine over the same time incubation, indicating highly efficient uptake of our nano‐vaccine by BMDCs (Figure 2E,F and Figure S8, Supporting Information). The efficient uptake by BMDCs might effectively enhance the cross‐presentation efficiency of neoantigen. To confirm this assumption, the immuno‐stimulation effects of BMDCs by nano‐vaccine were investigated. The BMDCs treated with equivalent doses of free CpG‐ODN or/and neoantigen were set as control groups. PBS treatment was set as the negative control, and LPS treatment was set as the positive control. Compared to the groups treated with free CpG‐ODN or/and neoantigen, the thiolated nano‐vaccine could elicit higher maturation of BMDCs, as indicated by the expression levels of co‐stimulatory receptors CD80+ and CD86+ (Figure 2G). To evaluate the in vitro immuno‐stimulation of BMDCs by our nano‐vaccine, the cytokines released by BMDCs were tested by EILSA. As shown in Figure 2H,I, the levels of interleukin‐12 (IL‐12) and tumor necrosis factor‐*α* (TNF‐*α*) detected in BMDCs treated with thiolated nano‐vaccine were higher than those in control groups, which benefited from the lysosome‐bypassing internalization of the thiolated nano‐vaccine. Thereafter, the matured BDMCs could further activate and promote T cell proliferation after 48 h co‐incubation (Figure 2J and Figure S9, Supporting Information). These results substantialized that the fast, highly efficient cytosol delivery of CpG‐ODN adjuvant and neoantigen could effectively activate BMDCs, and subsequently augment T cell proliferation.

### 2.3 In Vivo LN Homing and Immuno‐Stimulation Effects

**Figure 3:**
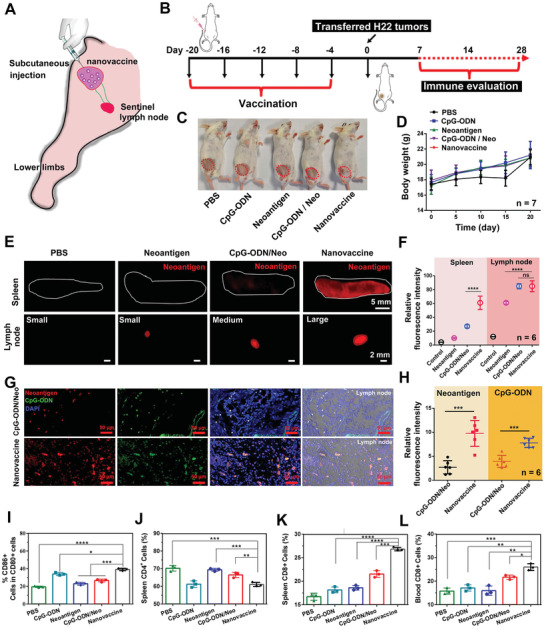
In vivo immuno‐stimulation and inguinal lymph node homing of thiolated nano‐vaccine. A) The immuno‐stimulation of thiolated nano‐vaccine in the left leg groin of mice. B) Schematic illustration of the nano‐vaccine inoculation protocol. C) Representative photo‐images of mice immunized with CpG‐ODN, neoantigen, CpG‐ODN/neoantigen mixture, and thiolated nano‐vaccine. D) Inoculated mice weight changes during the whole measurement days. Results are shown as mean ± SD. (*n* = 7). The fluorescence images of E,F) ex spleen and G,H) lymph node and relative fluorescence intensity calculated by ImageJ software. CLSM images and relative fluorescence intensity of LN slices after inoculated with neoantigen, CpG‐ODN/neoantigen mixture, and thiolated nano‐vaccine, at the 20th d respectively. Results are shown as mean ± SD. (*n* = 6). I) Induced DC maturation in tumor‐draining lymph nodes after inoculated with CpG‐ODN, neoantigen, CpG‐ODN/neoantigen mixture, and thiolated nano‐vaccine at the 20th d. The J) CD4+T cells and K) CD8+T cells in spleen or L) blood. The statistical analysis was performed with ANOVA analysis. Results are shown as mean ± SD. *p < 0.05, **p < 0.01, ***p < 0.001 (*n* = 3).

Next, the in vivo LNs homing property of thiolated nano‐vaccines was investigated after i. p. injection (in the left groin) for five times per 4 d (**Figure** **3**A–C). All procedures of animal studies were approved by the Ethics Committee for Animal Research of Mengchao Hepatobiliary Hospital of Fujian Medical University. The LNs and spleens were then extracted after immunization for 21 d, during which the body weight of mice in all groups was also measured to primarily evaluate the bio‐safety. As shown in Figure 3D and Figure S10, Supporting Information, neither significant body weight fluctuation nor accidental death happened, indicating the good bio‐safety of the nano‐vaccine. Then, the extracted LNs and spleen were imaged to evaluate the lymphoid organ homing effect of neoantigens. As shown in Figure 3E,F, the strongest red fluorescence of neoantigen^Cy3^ was observed in lymphoid organs such as LNs and spleen after treatment with the thiolated nano‐vaccine, demonstrating excellent homing ability of the nano‐vaccine to LNs. To confirm above observation, the LNs were further sliced and analyzed by CLSM (Figure 3G,H), where significantly stronger green (CpG‐ODN^FAM^) and red (neoantigen^Cy3^) fluorescence signals were observed for group treated with the thiolated nano‐vaccine. Noteworthy, the size of LNs after thiolated nano‐vaccine inoculation was also significantly larger than the LNs in all control groups, suggesting an efficient elicitation of immune response in vivo. Then, the maturation of DCs in LNs and the activation degree of T cells in spleen and blood were studied. As shown in Figure 3I–L and Figure S11, Supporting Information, the highest maturation percentage of DCs (CD80+/CD86+) in LNs, and the highest activation degree of CD8+T cells in spleen and blood were observed with thiolated nano‐vaccine inoculation. These findings suggested the effective activation of immune responses in mice by our thiolated nano‐vaccine, which would be promising for inhibiting H22 tumor growth.

### 2.4 In Vivo Prevention of Tumor Growth and Prolonging the Survival

**Figure 4:**
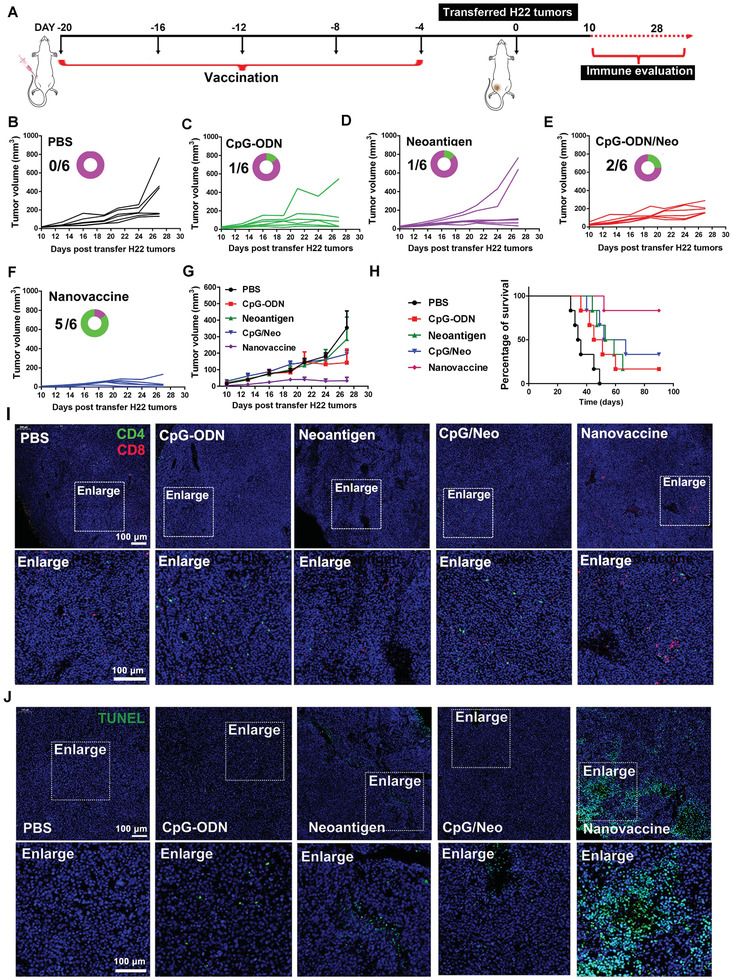
In vivo prevention of H22 tumor growth and prolonging the survival rate of H22 tumor‐bearing mice. A) Schematic illustration of inoculation procedure of thiolated nano‐vaccine. B–G) The tumor volume changes in H22‐tumor bearing BALB/c mice after inoculation with PBS, CpG‐ODN, neoantigen, the mixture of CpG‐ODN and neoantigen, and the thiolated nano‐vaccine for five times per 4d (the circular ring and number represent the survival of mice). H) Survival curve of H22 tumor‐bearing mice (*n* = 6 for all groups) after received different treatment as indicated. I) CLSM images of TILs inside the tumors at the 20th d after received different treatment as indicated. Red fluorescence represented CD8+T cells. Green fluorescence represented CD4+T cells and blue fluorescence represented nucleus. J) TUNEL staining of tumor slices at the 20th d after H22 tumor cell transfer.

Encouraged by the above results, the tumor prevention effects of thiolated nano‐vaccine in H22 tumor‐bearing (≈25 mm^3^) BALB/c mice were investigated. First, the mice inoculated with the thiolated nano‐vaccine in the left groin for five times per 4 d. After immunization, the tumor volume was measured by Vernier caliper after transferring of H22 tumors (≈25 mm^3^) (**Figure** **4**A). As shown in Figure 4B–G, the mice inoculated with PBS, CpG‐ODN, or neoantigen alone still suffered from rapid tumor growth. In contrast, the mice inoculated with CpG‐ODN/neoantigen mixture could delay tumor growth at certain degree, but still could not effectively prevent H22 tumor progression. However, the prevention of tumor growth after inoculation with our thiolated nano‐vaccine was highly efficient, and eventually prevented the tumor growth of 2/6 mice at 27th d. After observation for 90 d, 5/6 of mice with efficient prevention of tumor growth were achieved after thiolated nano‐vaccine inoculation as compared with PBS (0/6), CpG‐ODN (1/6), neoantigen (1/6) and CpG‐ODN/neoantigen (2/6) inoculated mice, respectively (Figure 4H and Figure S12, Supporting Information). The efficient tumor prevention effect of our thiolated nano‐vaccine was mainly attributed to the LN/spleen homing (Figure 4E–H), and efficient TILs infiltration into tumor tissues. To further confirm this assumption, tumors on the 20th day after H22 tumor transfer were excised, sliced, stained, and imaged by CLSM. As shown in Figure 4I, the higher frequency of CD8+T cells (red fluorescence) and lower frequency of CD4+T cells (green) were obviously observed after thiolated nano‐vaccine inoculation comparing to other groups, indicating the highly efficient CD8+ effector T cell infiltration, which could lead to strong antitumor immune responses to against H22 tumors. The results were further confirmed by the relatively higher amounts of CD69+/CD25‐ TILs on the tumor slices after thiolated nano‐vaccine inoculation (Figure S13, Supporting Information); the results were then proved by TUNEL staining of excised tumor slices. As shown in Figure 4J, a significantly higher amount of apoptotic/necrotic cells with strong green fluorescence could be also obviously observed after thiolated nano‐vaccine inoculation. These findings suggested our prepared thiolated nano‐vaccine could effectively prevent H22 tumor growth and prolong the survival time of inoculated mice through effective effector cell infiltration and activation of the CD8+T dependent immune responses.

### 2.5 Elimination of Primary Tumors and Prevention of Tumor Re‐Challenge

**Figure 5:**
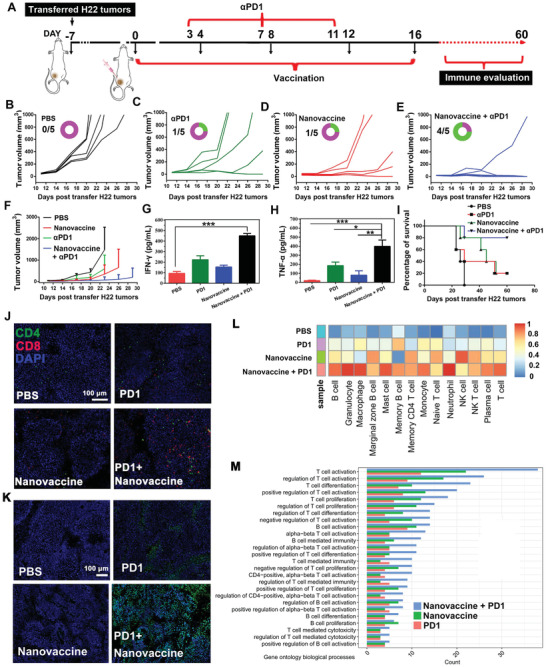
In vivo elimination of established primary tumors and prevention of tumor re‐challenge. A) Schematic illustration of thiolated nano‐vaccine inoculation procedure and PD‐1 antibody administration procedure in the established H22 tumor‐bearing mice. B–F) The tumor volume changes in H22‐tumor bearing BALB/c mice after thiolated nano‐vaccine inoculation for five times per 4 d or/and *α*PD1 for three times per 4 d, respectively. The PBS treated mice were set as the control. H) The tumor volume change along with time in each treated group. The secretion of G) IFN*γ* and H) TNF‐*α* in tumors. The statistical analysis was performed with ANOVA analysis. Results are shown as mean ± SD. *p < 0.05, **p < 0.01, ***p < 0.001 (*n* = 5). I) Survival curves of the H22 tumor‐bearing mice (*n* = 5 for all groups) after immunization and treatment as indicated. J) CLSM images of tumor‐infiltrating lymphocytes (TILs) in tumor tissues at the 20th d after receiving different treatment as indicated. Red fluorescence was CD8+T cells. Green fluorescence was CD4+T cells. K) TUNEL staining of tumor slice at the 20th d after received different treatment as indicated. L) Heat map analysis of ex tumors after receiving different treatment as indicated through Transcriptome Sequencing (RNA‐seq). Scale bar of 1 represents the total percentage of samples as indicated. M) Gene ontology biological process analysis of ex tumors after received different treatment as indicated.

Next, we evaluated the elimination of established primary H22 tumor through inoculation of thiolated nano‐vaccine. BALB/c mice were first subcutaneous transplanted with H22 tumor (≈25 mm^3^) (**Figure** **5**A). When the tumor size reaches ≈40 mm^3^, the mice were inoculated with the thiolated nano‐vaccine for five times per 4d. Moreover, to relieve the immunosuppression micro‐environment in tumors and augment the CD8+T cell‐dependent immunity, the immune checkpoint inhibitor *α*PD‐1 (2.5 mg kg^−1^) was contemporarily intravenously administrated to the mice at the tenth d after transfer of H22 tumors and sustained for three times per 4 d. PBS treated mice underwent a rapid tumor growth and the tumor volume reached above ≈1000 mm^3^ within only in 23 d (Figure 5B–F). In contrast, the tumors were suppressed after inoculation of thiolated nano‐vaccine alone to certain degree, but tumor growth still remained. This might due to overexpression of PDL1 in H22 tumors, which impeded the antitumor effects of T cell responses.^[^ ^37^ ^]^ However, by blocking immune checkpoint through systemic administration of *α*PD1 and i. p injection of thiolated nano‐vaccine, the tumor growth of H22 tumor was obviously suppressed, and it eventually eliminated the tumor of 4/5 mice at the 29th d comparing to PBS groups (0/5), *α*PD1 (1/5) or thiolated nano‐vaccine (1/5) treatment alone. These findings suggested that combination of *α*PD1 and thiolated nano‐vaccine could effectively suppress tumor growth and eliminate H22 tumors. The highly efficient cancer immunotherapeutic outcome of combining *α*PD1 with thiolated nano‐vaccine might be associated with the high accumulation of TILs in tumors. Then, the CD8+T cell infiltration in tumors were evaluated by FACS (Figure S14, Supporting Information). As expected, higher percentage of infiltrated CD8+T cells while lower percentage of infiltrated CD4+T cells in tumor of the combination strategy were clearly observed comparing to the PBS, *α*PD1 or thiolated nano‐vaccine treatment alone. In addition, higher levels of pro‐inflammation cytokines (TNF*α* and IFN*γ*) inside tumors were also observed by ELISA assay, indicating a robust antitumor immune response in vivo (Figure 5G,H), which led to the prolonged survival above 60 d by the combinational strategy (Figure 5I and Figure S15, Supporting Information). Moreover, higher amounts of TILs with the expression of CD8+/CD69+/CD25‐ in ex tumor slices were further visualized in the mice treated by the combinational strategy through immunofluorescent staining and immunohistochemical analysis (Figure 5J and Figure S16, Supporting Information); meanwhile, the cancer cell killing effects were further confirmed by TUNEL staining (Figure 5K). Previous studies have shown that the number and activation status of tumor infiltrating immune cells were significantly related to the immunotherapy efficacy, which could indicate the prognosis of cancer patients.^[^ ^38^ ^]^ When there are significantly increased numbers of TILs in tumor tissues, it indicates that the body has initiated a strong immune response against tumors.^[^ ^38^, ^39^ ^]^ Therefore, the antitumor molecular mechanisms were further briefly investigated. As shown in Figure 5L,M, the heat map analysis from the tumors’ transcriptome data showed excellent immune effector cell accumulation in tumors including B cells, granulocyte, macrophage, memory CD4+T cells, NK cells, NK T cells, and T cells after the combinational strategy of ICB and nano‐vaccine treatment; gene ontology (GO) biological process analysis further confirmed above results, displayed robust immune responses against H22 tumors after the combination treatment.

**Figure 6:**
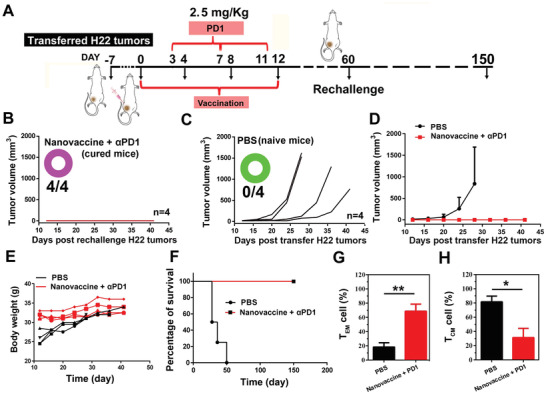
In vivo prevention of H22 tumor re‐challenge and prolonging the survival of H22 tumor bearing mice after re‐challenge. A) Schematic illustration of the thiolated nano‐vaccine inoculation procedure, anti‐PD‐1 antibody administration procedure, and re‐challenge procedure. B–D) The H22 tumor re‐challenge in BALB/c mice with PBS treatment (*n* = 4) and cured mice by the combination of *α*PD‐1 and nano‐vaccine (*n* = 4). E) Body weight of mice during the treatment as indicated, (*n* = 4). F) Survival curves of the H22 tumor‐bearing mice (*n* = 4 for all groups) after indicated treatment. G) The *T*_EM_ cell percentage and H) the *T*_CM_ cell percentage in LNs after receiving different treatment as indicated. The statistical analysis was performed with the two‐tail paired Student's *t*‐test. Results are shown as mean ± SD. *p < 0.05, **p < 0.01 (*n* = 4).

Furthermore, the efficient antitumor immune responses might also prevent tumor re‐challenge. Thereafter, the cured mice were further transferred with H22 tumors (≈25 mm^3^) at the 60th d after the first nanovaccine injection, and then the tumor volume was evaluated by Vernier caliper (**Figure** **6**A). As shown in Figure 6B–D, it was amazing that the transferred H22 tumors could not grow anymore in these cured mice (*n* = 4), but not in the PBS treated naive mice where tumors underwent a relative rapid growth. Besides, during the treatment for 40 d, no obvious weight fluctuation happened, suggesting the limited side effects to mice (Figure 6E). After observing for 150 d, the efficient prevention of tumor re‐challenge with long‐term survival above 150 d in cured H22 tumor bearing mice was clearly observed, but not in the PBS treated mice which only had limited survival of 50 d, suggesting the long‐term immune memory effects to efficiently against cancer (Figure 6F and Figure S17, Supporting Information). Therefore, the percentages of systemic central memory T cells (*T*_CM_) and effector memory T cells (*T*_EM_) in LNs were then measured. The results showed that the highest frequency of CD8+*T*_EM_ and CD4+*T*_EM_ while the lowest frequency of the CD8+*T*_CM_ and CD4+*T*_CM_ were observed in the cured mice, demonstrating the ability of the combinational therapeutic strategy to effectively turn CD8+*T*_CM_ and CD4+*T*_CM_ into a TEM phenotype, which could provide more‐potent antitumor immune memory responses to protect against tumor re‐challenge (Figure 6G,H and Figure S18, Supporting Information). Moreover, after combination strategy treatment for 150 d, there were no abnormal index in ALT, AST et al., biochemical indexes suggested the bio‐safety of this combination strategy (Figure S19, Supporting Information). These findings demonstrated that our prepared nano‐vaccine could combine with ICB for strengthening the T cell immunity to eliminate the H22 tumors, prevent tumor re‐challenge and prolong survival of mice, which might hold great promise for personalized immunotherapy of HCC.

## 3 Discussion

Various nano formulations have been developed to design cancer vaccine through payload peptide/adjuvant such as PLGA, micelles, MSN, and liposome, and have been proved to enhance the immunogenicity of peptide‐based cancer immunotherapy;^[^ ^40^, ^41^, ^42^ ^]^ however, the severe degradation of peptide and adjuvant (TLR9) significant impairs the cross‐presentation efficiency of APCs and antitumor T cell immunity, and it remains to be improved.^[^ ^43^, ^44^ ^]^ The work presented here introduced a versatile methodology to design cancer nano‐vaccine through simply mixing and shaking of Gu^+^‐unit and CpG‐ODN/neoantigen for only 15 min, and the obtained nano‐vaccine showed a relative uniform nanosize and limited drug leakage (Figure 2B and Figure S6, Supporting Information). This simple formulation may be more reliable and easier for quality control for clinical translation. The body weight and blood biochemical analysis suggest the low toxicity and potential of bio‐safety of our prepared nano‐vaccine. By taking advantage of the thiolated‐mediated cytosolic delivery strategy, our prepared nano‐vaccine can more efficiently enhance the local concentration to significantly augment the presentation efficiency by APCs both in vitro and in vivo (Figure 2G–J). After inoculation of nano‐vaccine into BALB/c mice, the efficient LN homing and payload neoantigen uptake by APCs lead to abundant TIL infiltration in tumors (Figure 4J). Then, it can induce a robust CD8+T cell immune response to effectively suppress tumor growth and prolong the survival of 83% mice (Figure 4G,H).

With a view to the tumor immunosuppression micro‐environment, especially in solid tumors, here we provide an essential insight to significantly improve the therapeutic efficiency of thiolated nano‐vaccine through combining with ICB on the T cells via systematic administration of *α*PD1. Accordingly, relieving the immunosuppression microenvironment leads higher levels of inflammatory factors (IFN‐*γ* and TNF‐*α*) in tumor tissues and stronger antitumor immune responses, which significantly enhanced the elimination efficiency of the established H22 tumors in mice (Figure 5A–L). Meanwhile, the mechanism study was further conducted by GO biological process analysis, and effective immune effector cell accumulation in tumors after the combinational strategy of ICB and nano‐vaccine treatment are revealed, which further confirmed above explanations (Figure 5L,M). Furthermore, the excellent immune memory effects might significantly contribute to the prevention of tumor re‐challenge (Figure 6). Thus, enhancement of direct cytosolic delivery efficiency of PCVs and regulation of T cells through ICB may provide a new sight to effectively treat HCC tumors.

## 4 Experimental Section

##### Materials

All oligonucleotides and CpG‐ODN (TCC ATG ACG TTC CTG ACG TT‐FAM or TCC ATG ACG TTC CTG ACG TT) were synthesized and purified by HPLC in Sango Biotechnology Co., Ltd (Shanghai, China). Neoantigens (HTDAHAQAFAALFDSMH) were synthesized and purified by HPLC in GenScript USA Inc. Antibodies used in this study were purchased from BioLegend, Inc. (San Diego, USA). LysoTracker Green and Hoechst 33342 were obtained from Thermo Fisher Scientific Inc (USA). *α*PD1 was purchased from BioLegend, Inc (USA). ELISA Kits for IFN‐*γ* and TNF‐*α* were purchased from Neobioscience Technology (Shenzhen, China). Unless specified, all other chemicals were commercially available and used as received. Mouse liver cancer cells (H22) were cultured in RPMI‐1640 medium containing 10% fetal bovine serum, 100 IU mL^−1^ of penicillin, and 100 µg mL^−1^ of streptomycin.

##### Assembly and Characterization of Thiolated Nano‐Vaccine

To prepare thiolated nano‐vaccine, guanidinium (Gu^+^)‐containing disulfide monomer was first synthesized according to the previous publication.^[^ ^34^ ^]^ Then, 500 nm of 20 nt CpG‐ODN and 16.45 µm of neoantigens were mixed with 16.45 µm of Gu^+^‐containing disulfide monomers for 15^ ^min at room temperature in TM buffer (20 × 10^−3^ m Tris, 10 × 10^−3^ m and MgCl_2_, pH 7.5). Subsequently, the un‐assembled CpG‐ODN, neoantigens, and disulfide monomers were removed by dialysis (MWCO 3.5 kDa) in water for 24 h. The thiolated nano‐vaccine was obtained.

##### Apparatus

Scanning electron microscope (SEM) was performed using the Nova NanoSEM 230 (USA) to characterize the morphology and nano size of thiolated nano‐vaccine. Nano ZS (Malvern Instruments, Malvern UK) was used to investigate the DLS of thiolated nano‐vaccine. Zeta potential measurements were also performed at 25 °C on the Nano ZS. The Uv–vis‐NIR absorbance was performed by a Vis‐NIR spectrometer (Spectro Max M5e, Germany). Flow cytometric analysis was performed using a BD FACS II (USA).

##### PAGE Assay

PAGE was performed to evaluate the assembly of thiolated nano‐vaccine. Briefly, 10 µL nano‐vaccine was loaded onto 6% polyacrylamide gel with 1 µL 6 × loading buffer after shaking the mixture of CpG‐ODN and thiolated neoantigen with disulfide monomers for 1, 3, 5, 10, and 15 min. After electrophoresis at 100 V for 20 min, the gels were stained by GelRed and analyzed by ChemiDoc MP Imaging System from Bio‐Rad. To evaluate the stability of thiolated nano‐vaccine, it was incubated with 1 mg mL^−1^ BSA at 37 °C for 24 h, and then analyzed by PAGE.

##### CLSM Imaging of Thiolated Nano‐Vaccine in BMDCs

The BMDCs were obtained from health BALB/c male mice, and cultured in RPMI 1640 medium. Afterward, the immature BMDCs were co‐incubated with nano‐vaccine which was assembled by FAM labeled CpG‐ODN, Cy3 labeled neoantigen, and disulfide monomers for 0.5 h. To investigate the subcellular localization of nano‐vaccine, the BMDCs were first stained by LysoTracker Green (50 nm) and then incubated with the nano‐vaccine that was assembly by CpG‐ODN, Cy3 labeled neoantigen and disulfide monomers for 0.5 h or 2 h, respectively. The treated cells were then imaged by confocal microscope (CLSM) (LSM 780, USA) with 488 nm laser excitation for LysoTracker Green and 561 nm laser excitation for Cy3, respectively.

##### BMDC Maturation and T Cell Proliferation

The isolation and detection of BMDCs from mouse bone marrow were performed according to the previously reported work.^[^ ^33^ ^]^ The tibias and femurs were isolated from mice and kept in RPMI 1640 medium on ice, afterward the end of each bone was cut off with scissors, and the cells were flushed out from the bone by RPMI 1640 culture medium through a syringe. The cell containing medium was passed by 40 µm cell trainer to remove debris. Then the cells were collected by centrifugation at 800 × *g* for 5 min, followed by suspending in red blood cell lysis buffer for 10 min to lyse red blood cells. Afterward, the cells were washed with medium through centrifuging by 5 min at 800 × *g*, then seeded in a 6‐well plate with medium and cultured with mouse granulocyte/macrophage colony stimulating factor (mGM‐CFS, 20 ng mL^−1^) and IL‐4 (10 ng mL^−1^) for 5 d. Subsequently. The immature BMDCs were then co‐incubated with CpG‐ODN, neoantigen, CpG‐ODN/neoantigen mixture (CpG‐ODN/Neo), and nano‐vaccine for 48 h. LPS was used as the positive control. The culture medium was collected for cytokine analysis through Mouse ELISA Kit. The treated BMDCs were stained with CD11c, CD80, and CD86 antibodies (eBioscience) and then analyzed by flow cytometry. To isolate the CD8+T cells from spleen, the spleen was first harvested from mice and kept in RPMI 1640 medium on ice. Then, the cells were flushed out from spleen by medium through a syringe. The cell containing medium was passed through 40 µm cell strainer to remove large debris. Afterward, the cells were collected by centrifugation at 800 × *g* for 5 min, followed by suspending in red blood cell lysis buffer for 10 min at room temperature to lyse red blood cells. Subsequently, the cells were washed with medium by centrifuging for 5 min at 800 × *g*. Then, the CD8+T cells were sorted through CD8 (TIL) MicroBeads (Miltenyi Biotec) according to the manufacturer's instructions. To further detect the T cell proliferation, the T cells (1 × 10^5^) were stained by 2 µm CFSE at 37 °C for 30 min. The CFSE‐labeled T cells were then mixed with the matured BMDCs for 72 h co‐incubation. Then, the T cell proliferation was examined by FACS. The detection indicators for matured BMDCs are expression of CD11c+/CD80+/CD86+, for the population of T cells are expression of CD3+/CD8+, for the memory T cells are expression of *T*_CM_ (CD44+/CD62L‐) and *T*_EM_ (CD44+/CD62L‐).

##### Draining LN and Spleen Homing of Thiolated Nano‐Vaccine

To investigate the LN and spleen homing, the BALB/c mice were subcutaneously inoculated with CpG‐ODN^FAM^, Neoantigen^Cy3^, CpG‐ODN^FAM^ + Neoantigen^Cy3^ mixture, or thiolated nano‐vaccine in the right groin for five times per 4 d, respectively. Afterward, the inguinal LNs were harvested, and imaged by ChemiDoc MP Imaging System from Bio‐Rad. Consequently, single cells isolated from LNs or spleen through collagenase D and DNase I pre‐treatment were collected and then stained with CD11c‐APC, CD80‐PE, and CD86‐PE‐Cy7 (eBioscience) for immune response analysis. The activated T cells that isolated from spleen and blood were stained with anti‐CD3‐APC, anti‐CD4‐FITC, anti‐CD8‐PE and analyzed by flow cytometry.

To analyze the memory T cells, the blood collected from the mice with thiolated nano‐vaccine treatment was diluted with PBS (blood volume: PBS volume = 1: 1) and subject to a density gradient centrifugation in Ficoll Paque TM PREMIUM sterile solution at 800 × *g* for 30 min at 4 °C. Afterward, the cells were harvested and then examined by flow cytometry after staining with anti‐CD4‐FITC, anti‐CD44‐PE‐Cy7, and anti‐CD62L‐PerCP‐Cy5.5 antibody or anti‐CD8‐PE, anti‐CD44‐PE‐Cy7, and anti‐CD62L‐PerCP‐Cy5.5 antibody (eBioscience).

##### In Vivo Prevention of Tumor Growth and Re‐Challenge Studies

To test the tumor growth prevention effects of the thiolated nano‐vaccine, male BALB/c mice (≈4–5 weeks old) were purchased from China Wushi, Inc. (Shanghai, China). All animal procedures were approved by the Animal Ethics Committee of Mengchao Hepatobiliary Hospital of Fujian Medical University. After subcutaneously inoculated with CpG‐ODN^FAM^, Neoantigen^Cy3^, CpG‐ODN^FAM^ + Neoantigen^Cy3^ mixture, or thiolated nano‐vaccine in the right groin for five times per 4 d respectively, the mice were then transferred with H22 tumors (25 mm^3^). Afterward, the tumor size was measured using caliper every other day after the treatment. The tumor volume (*V*) was calculated using the following equation: *V* = 1/2(*A* × *B* ^2^), where *A* and *B* are the longer and shorter diameter (mm) of the tumor, respectively.

To investigate the elimination efficiency of thiolated nano‐vaccine to the established tumors, the male BALB/c mice (5 weeks old) were first transferred with H22 tumors (≈25 mm^3^) (The expanded H22 cells from BALB/c enterocoelia were first collected by centrifuge. Afterward, the mice were then subcutaneously injected with H22 cells (5 × 10^6^ cells) in PBS buffer. When the tumor size was reached ≈7 mm (≈150 mm^3^), the ex vivo tumors were cut into small tumor pieces ≈25 mm^3^, and then subcutaneously transferred into mice through surgery). Afterward, these mice were then subcutaneously inoculated with thiolated nano‐vaccine (or controls including PBS, CpG‐ODN^FAM^, Neoantigen^Cy3^, and CpG‐ODN^FAM^ + Neoantigen^Cy3^ mixture) for five times per 4 d, or/and intravenously injected with *α*PD1 (2.5 mg kg^−1^) for three times per 4 d. The tumor volume (*V*) was calculated by using Vernier caliper. The TILs (CD4+/CD8+) were investigated by CLSM imaging. Briefly, the tumors were dissected after subcutaneous inoculation of thiolated nano‐vaccine (or controls including PBS, CpG‐ODN^FAM^ (8.8 µg mL^−1^), Neoantigen^Cy3^ (6.11 µg mL^−1^), CpG‐ODN^FAM^ + Neoantigen^Cy3^ mixture) for five times per 4 d, or/and intravenous injection of *α*PD1 (2.5 mg kg^−1^) for three times per 4 d (at 20th d), and then stained with CD8+CD4+ CD25+ antibodies. After observation for 60 d, the cured mice were then then further transferred with H22 tumors (≈25 mm^3^). The tumor volume (*V*) was calculated by using Vernier caliper.

##### Systemic Toxicity was Assessed by Body Weight Loss and H&E Staining

The long‐term systematic toxicity assessment was also performed. Briefly, the mice were subcutaneously inoculated with thiolated nano‐vaccine for five times per 4 d, or/and intravenously injected with *α*PD1 (2.5 mg kg^−1^) for three times per 4 d, respectively. The treated mice were sacrificed at the 20th or the 150th d after receiving various treatments as indicated, and the major organs (e.g. heart, liver and spleen et al.) of the treated mice were collected, then fixed in 4% neutral formaldehyde, and further stained with hematoxylin and eosin (H&E), and imaged by Zeiss microscope (Axio Lab.A1).

##### RNA Sequencing and Bioinformatic Analysis

RNA samples were extracted from tumor tissues after receiving indicated treatments and further subjected to RNA library preparation using VAHTS Stranded mRNA‐seq Library Prep Kit for Illumina V2 kits. Then, RNA‐seq was conducted on Illumina HiSeq X10 system at Fulgent. Co., Ltd. After that, the infiltration degree of different immune cell types in tumor samples after receiving indicated treatments were evaluated by single sample Gene Set Enrichment Analysis algorithm based on 14 immune gene sets according to the previous study.^[^ ^45^ ^]^ The packages (GSVA, GSEABase, and limma) provided by Bioconductor (www.bioconductor.org) were employed to analyze the data via R software (version 3.6.3). Immune‐related biological processes were analyzed by GO enrichment analysis based on differential gene expression profiles.

##### Statistical Analysis

Statistical analysis of data was analyzed through one‐way of variance (ANOVA) for comparison among multiple groups or the two‐tail paired Student's *t*‐test for comparison between 2 groups, *p < 0.05 was set as statistically significant. **p < 0.01, ***p < 0.001. All the data were shown as means ± SD through at least three experiments.

## Conflict of Interest

The authors declare no conflict of interest.

## Supporting information

Supporting Information
