# Supplementary material for: Cytosolic Delivery of Thiolated Neoantigen Nano‐Vaccine Combined with Immune Checkpoint Blockade to Boost Anti‐Cancer T Cell Immunity
Source: Adv Sci (Weinh). 2021 Jan 29;8(6):2003504. doi: 10.1002/advs.202003504 (PMC7967047; doi:10.1002/advs.202003504)
Supplement: Supplementary file 1 — Supporting Information [file ADVS-8-2003504-s001.pdf]

© 2020 Wiley-VCH GmbH

ADVANCED SCIENCE
Open Access

Supporting Information

for *Adv. Sci.,* DOI: 10.1002/advs.202003504

Cytosolic Delivery of Thiolated Neoantigen Nano-vaccine Combined with Immune Checkpoint Blockade to Boost Anti-cancer T Cell Immunity

Da Zhang, Ziguo Lin, Ming Wu, Zhixiong Cai, Youshi Zheng, Lei He, Zhenli Li, Jie Zhou, Liqin Sun, Geng Chen, Yongyi Zeng, Juan Li, Jingfeng Liu, Huanghao Yang,* Xiaolong Liu**

© 2020 Wiley-VCH GmbH

Supporting Information

Cytosolic Delivery of Thiolated Neoantigen Nano-vaccine Combined with Immune Checkpoint Blockade to Boost Anti-cancer T Cell Immunity

Da Zhang[1, 2†], Ziguo Lin[1†], Ming Wu[1,3†], Zhixiong Cai[1,3], Youshi Zheng[1,3], Lei He[1,3], Zhenli Li[1,3], Jie Zhou[2], Liqin Sun[2], Geng Chen[1,3], Yongyi Zeng[1,3], Juan Li[2], Jingfeng Liu[1,3,4*], Huanghao Yang[2]*, Xiaolong Liu[1,3,4]*

Da Zhang, Ziguo Lin, Dr. Ming Wu, Dr. Zhixiong Cai, Youshi Zheng, Lei He, Dr. Zhenli Li, Geng Chen, Yongyi Zeng, Prof. Jingfeng Liu, Prof. Xiaolong Liu
1. The United Innovation of Mengchao Hepatobiliary Technology Key Laboratory of Fujian Province, Mengchao Hepatobiliary Hospital of Fujian Medical University, Fuzhou 350025, P. R. China

Da Zhang, Jie Zhou, Liqin Sun, Prof. Juan Li, Prof. Huanghao Yang
2.The Key Lab of Analysis and Detection Technology for Food Safety of the MOE, Fujian Provincial Key Laboratory of Analysis and Detection Technology for Food Safety, College of Chemistry, Fuzhou University, Fuzhou 350002, P.R. China

Dr. Ming Wu, Dr.Zhixiong Cai, Youshi Zheng, Lei He, Dr. Zhenli Li, Geng Chen, Yongyi Zeng, Prof. Jingfeng Liu, Prof. Xiaolong Liu,
[3]Mengchao Med-X Center, Fuzhou University, Fuzhou 350116, P. R. China

Prof. Jingfeng Liu, Prof. Xiaolong Liu
[4]CAS Key Laboratory of Design and Assembly of Functional Nanostructures, Fujian Institute of Research on the Structure of Matter, Chinese Academy of Sciences, Fuzhou 350002, P. R. China

E-mail: xiaoloong.liu@gmail.com, drjingfeng@126.com, hhyang@fzu.edu.cn;

[†]These authors contributed equally to this work.

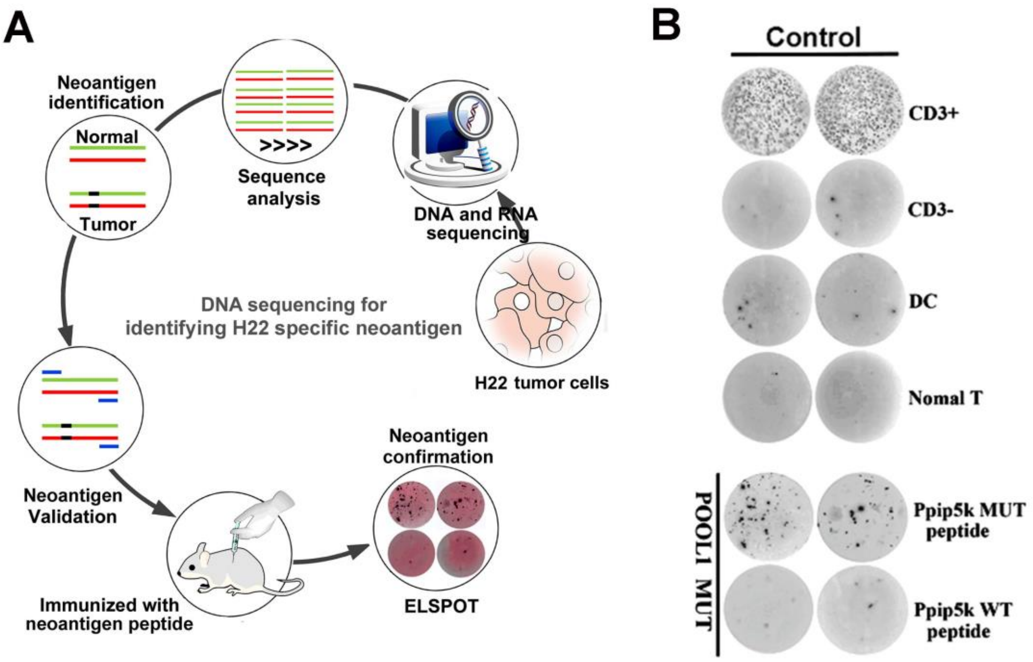

Figure. S1. Schematic depiction of H22 specific neoantigen screening for immunotherapy. The potential H22 cell (mouse liver cancer cell) specific neoantigens were screened by *in silico* analysis of whole-exome sequencing data and transcriptome sequencing data of H22 cells, and further confirmed by ELISPOT assay.

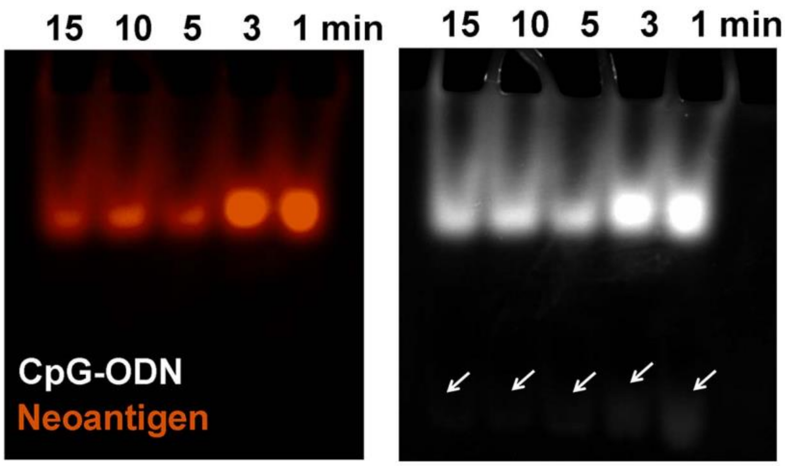

Figure. S2. Optimization of Gu+ monomer to CpG-ODN/Neoantigen reaction time by PAGE, and fluorescence image existed by 561 nm (right).

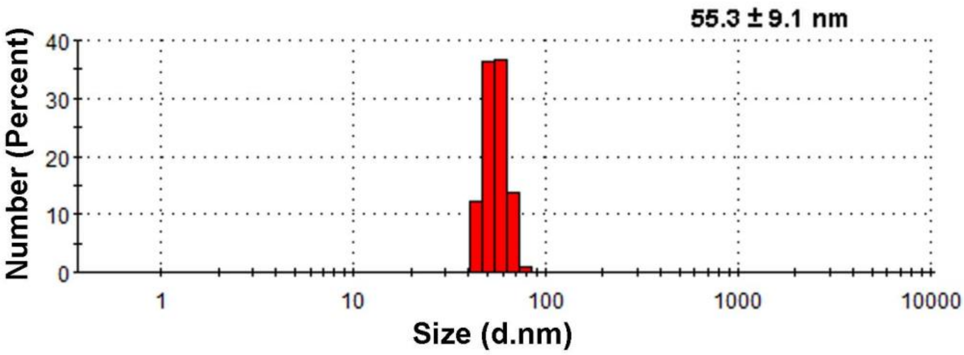

Figure. S3. DLS analysis of CpG-ODN NPs after self-assembly from CpG-ODN and Gu^{+} unit.

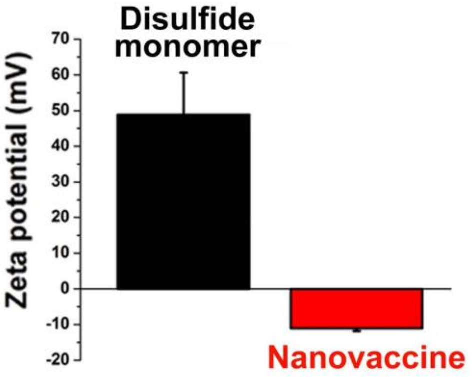

Figure. S4. Zeta potential of Gu^{+} unit and thiolated nano-vaccine.

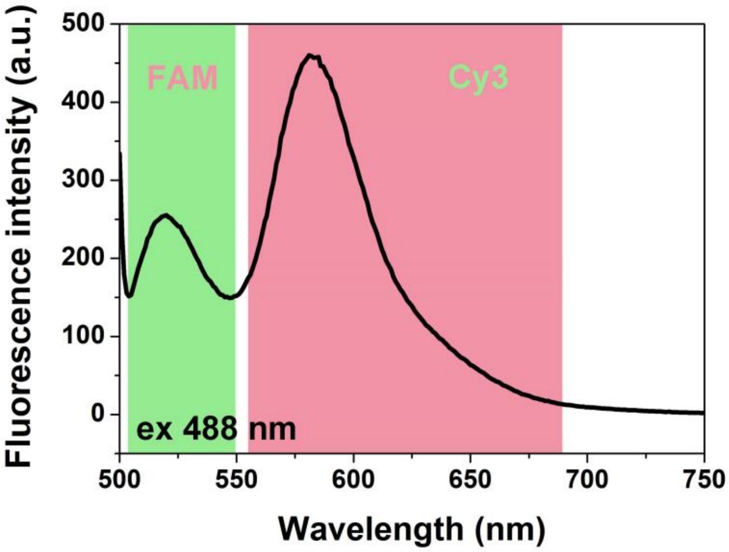

Figure. S5. Fluorescence spectrum of thiolated nano-vaccine (excited by 488 nm).

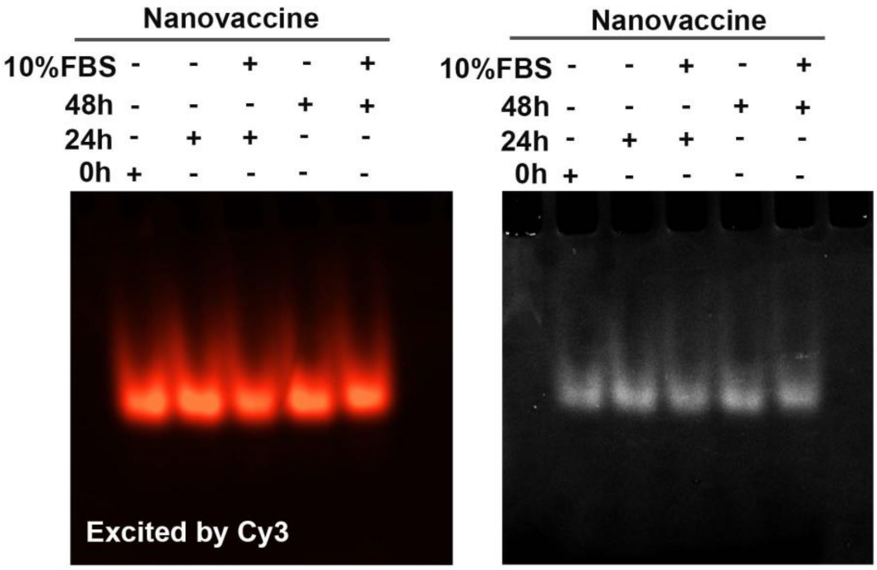

Figure. S6. PAGE profiles of thiolated nano-vaccine with or without incubation with 10% FBS at 4°C for 0, 24 and 48 h, respectively.

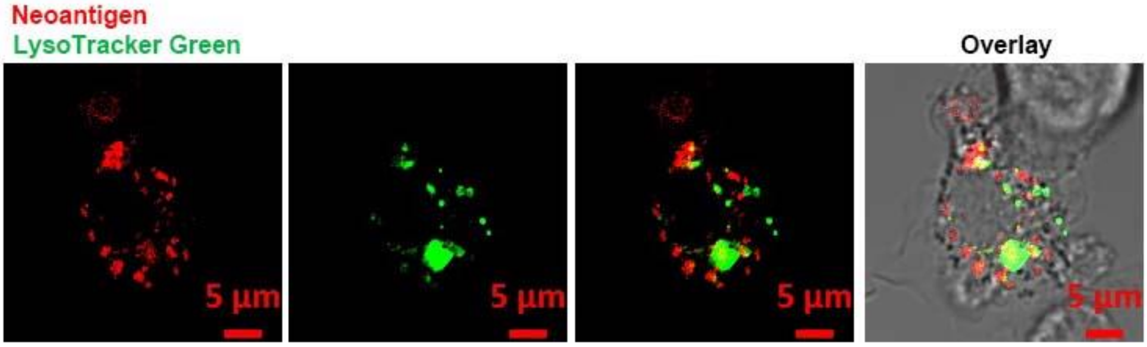

Figure. S7 The sub-cellular location of thiolated nano-vaccine (CpG-ODN / neoantigen^{Cy3}) in BMDCs after 2 h incubation and lysosome staining with LysoTracker Green.

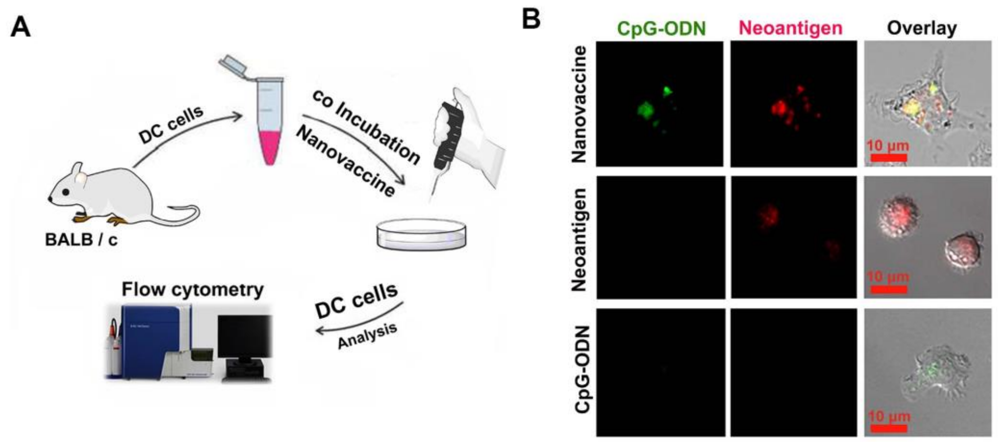

Figure. S8. CLSM images of BMDCs after co-incubation with CpG-ODN^{FAM}, neoantigen^{Cy5} or thiolated nano-vaccine (CpG-ODN^{FAM} and neoantigen^{Cy5}) for 0.5 h, respectively.

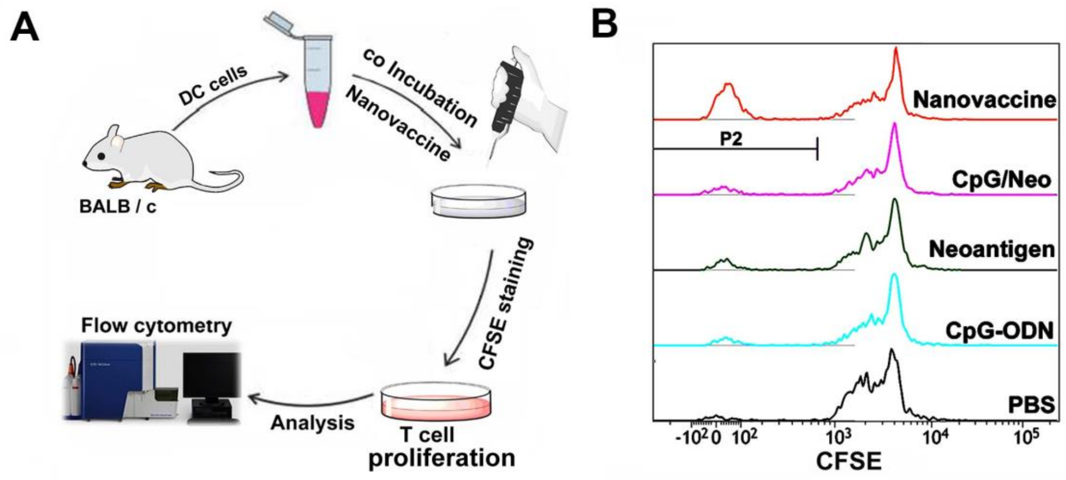

Figure. S9. Analysis the CD8+T cell proliferation by CFSE after co-incubation with matured BMDCs that received different treatments as indicated for 72 h, respectively.

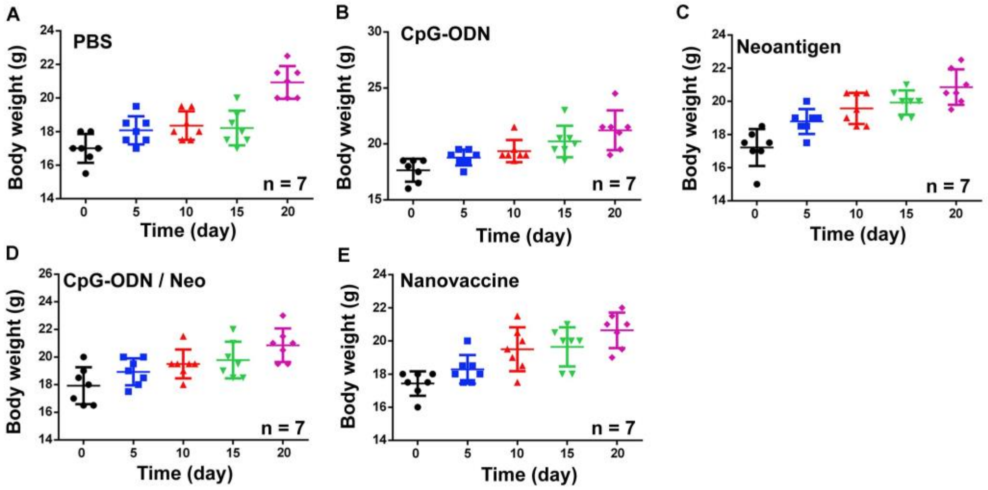

Figure. S10. (A to E) Body weight changes of the H22 tumor bearing mice with indicated treatments (n = 7).

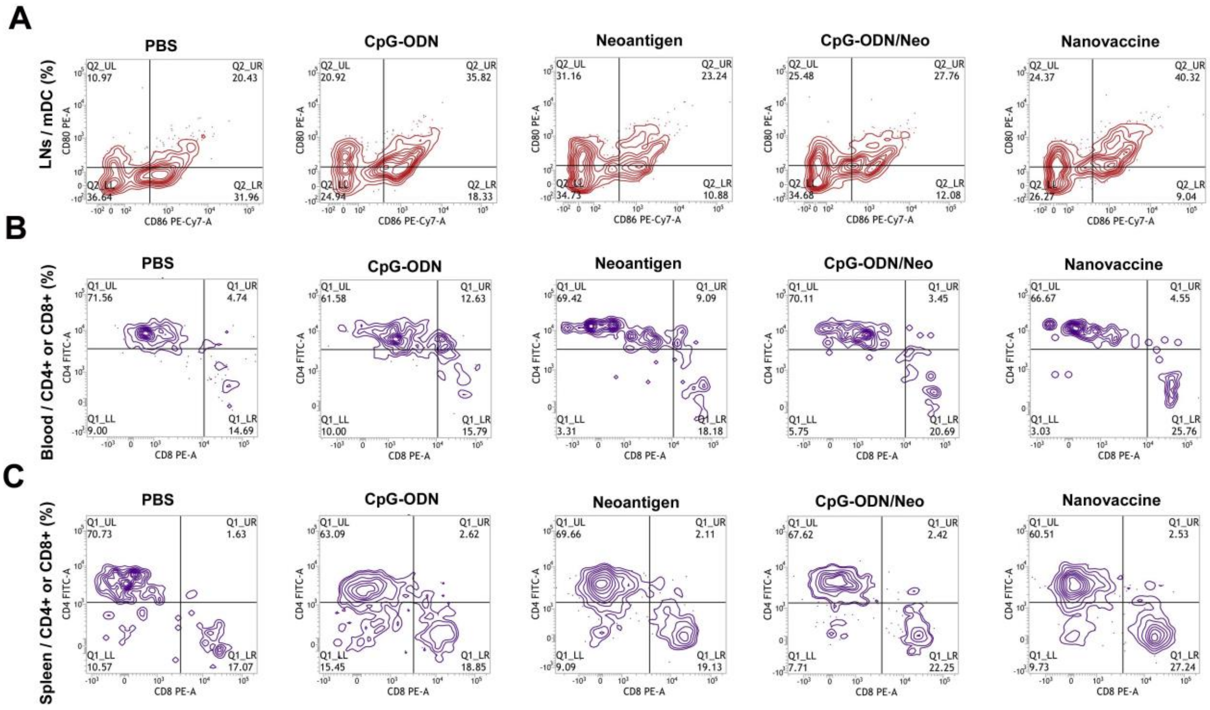

Figure. S11. (A) induced DC maturation in tumor-draining lymph nodes on BALB/c mice after inoculation of PBS, CpG-ODN, neoantigen, CpG-ODN/neoantigen mixture and nano-vaccine (gated on CD11c+DC cells). Cells in the tumor-draining lymph nodes were collected at the 21th d after indicated treatments for FACS assessment after staining with CD11c, CD80 and CD86. (B) The blood and (C) spleen of CD4+ / CD8+T lymphocytes were examined by FACS after indicated treatments.

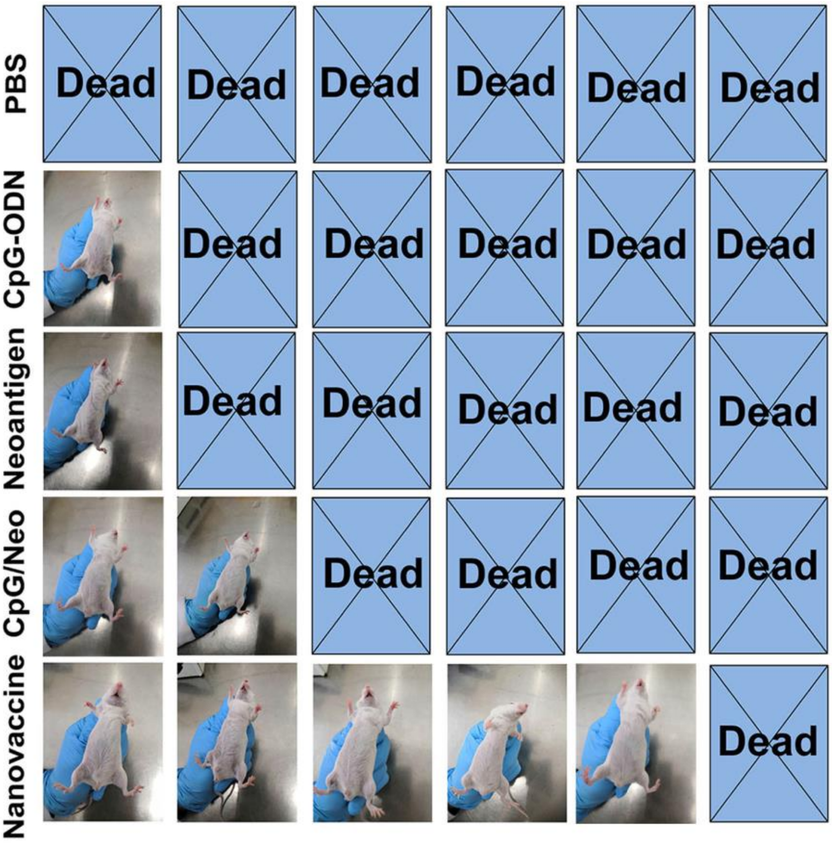

Figure. S12. Photo images of survived mice with inhibition of tumor growth in each group at the 90th d.(n = 6).

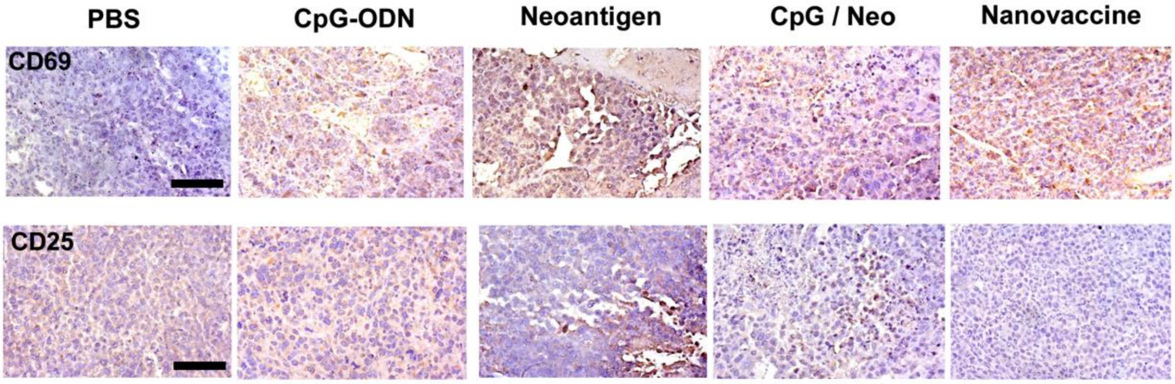

Figure. S13. IHC staining of the tumor slices to analyze the infiltrated CD69 and CD25 positive cells after receiving different treatment as indicated. Scale bar, 100 μm.

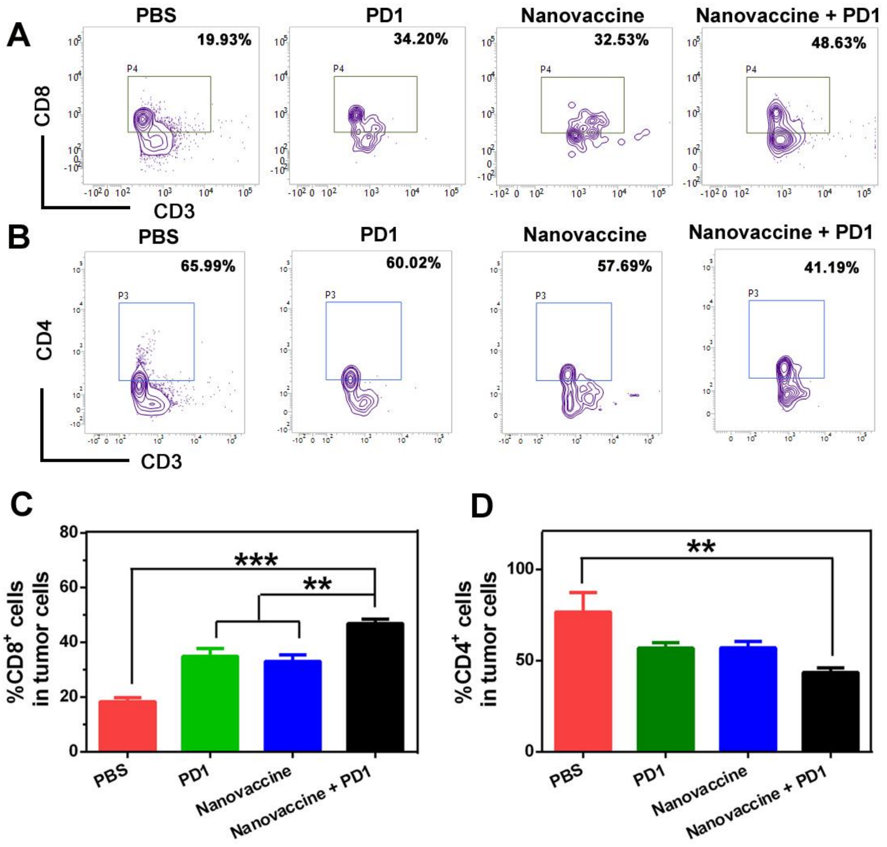

Figure. S14. (A to D) CD4+ / CD8+ T lymphocytes in H22 tumors after receiving indicated treatment which was measured by FACS after staining with CD4 and CD8. The statistical analysis was performed with ANOVA analysis. Results are shown as mean ± SD. ***p* < 0.01, ****p* < 0.001 (n =5).

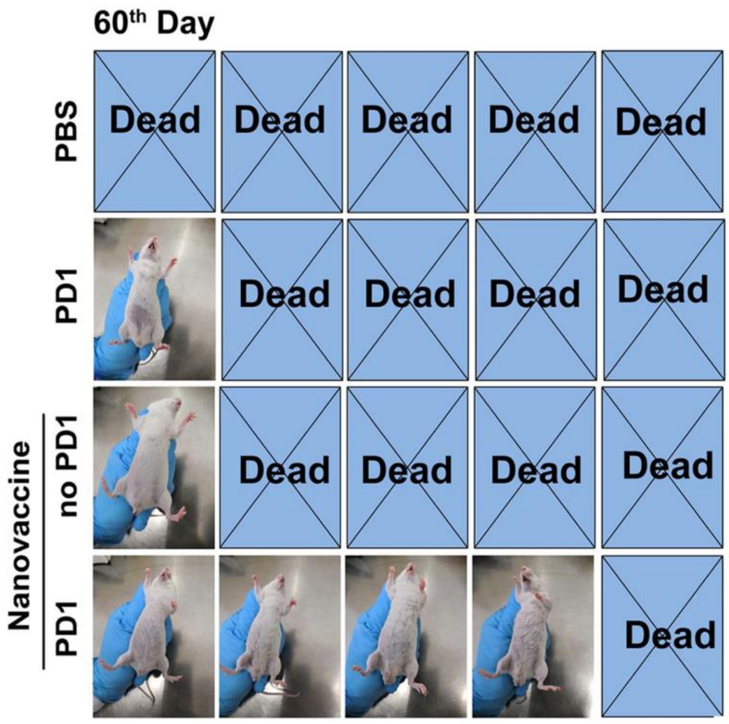

Figure. S15. Photo images of survived mice with inhibition of tumor growth in each group. (n = 5).

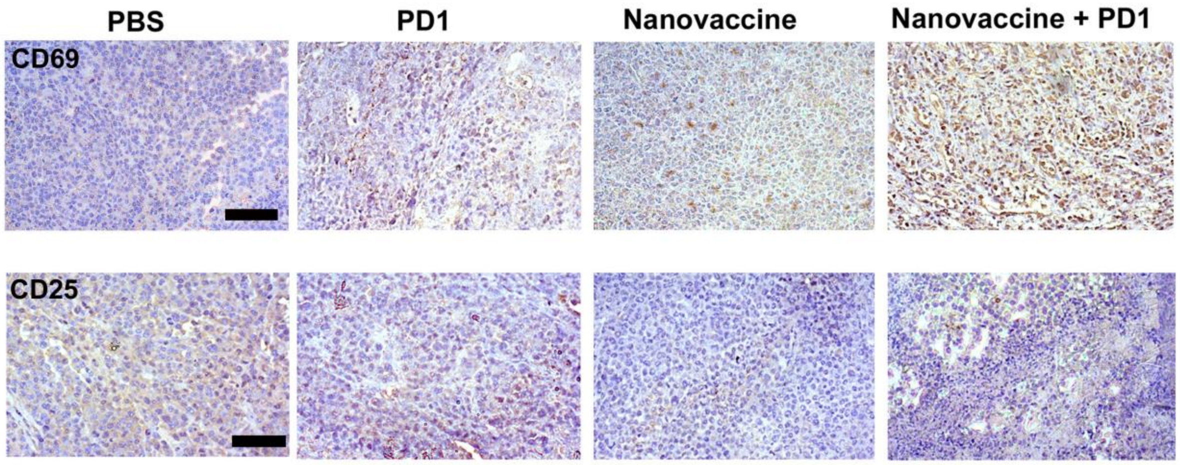

Figure. S16. IHC staining of the tumor slices to analyze the infiltrated CD69 and CD25 positive cells after receiving different treatment as indicated. Scale bar, 100 μm.

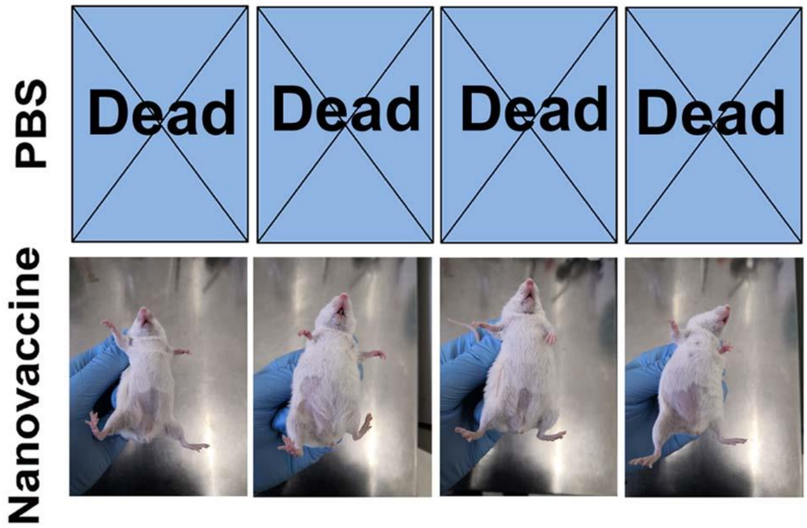

Figure. S17. The photo images of survived mice with prevention of tumor growth after re-challenge. (n = 4).

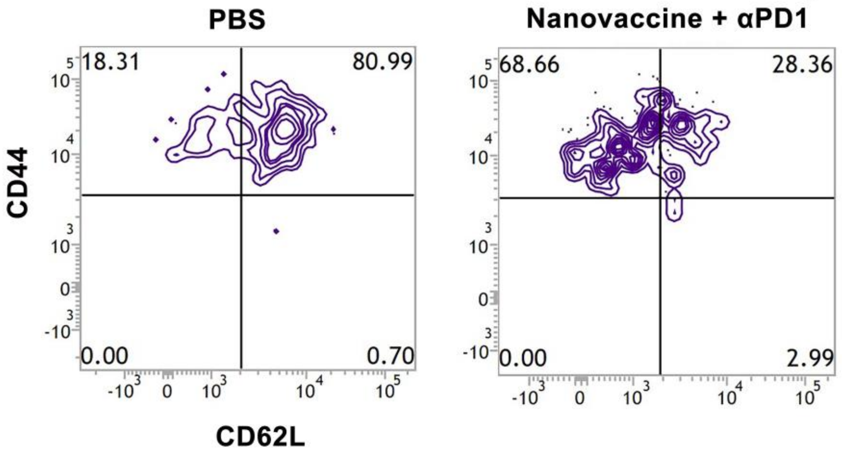

Figure. S18. FACS scatter plots of the T_{CM} (CD44+/CD62L^{-}) and T_{EM} (CD44+/CD62L^{-}) in tumor-draining LNs isolated from sacrificed mice after various treatments by staining with CD44 and CD62L antibodies.

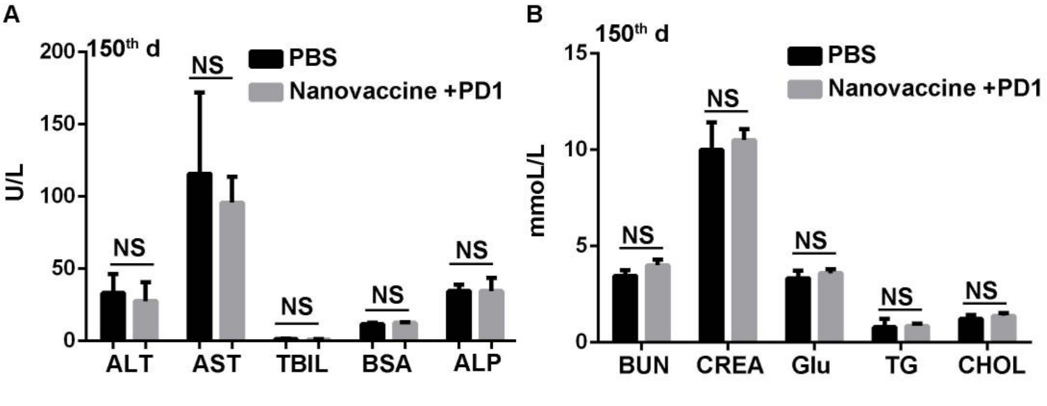

Figure. S19. Blood biochemistry analysis of liver (A) and kidney (B) function markers in H22 tumor bearing mice after combination strategy treatment for 150 d. Results are shown as mean ± SD. (n=4). The statistical analysis was performed with ANOVA analysis, NS presented as no significance.
